# Functional validation of *EIF2AK4* (GCN2) missense variants associated with pulmonary arterial hypertension

**DOI:** 10.1093/hmg/ddae082

**Published:** 2024-05-22

**Authors:** Giulia Emanuelli, JiaYi Zhu, Wei Li, Nicholas W Morrell, Stefan J Marciniak

**Affiliations:** Cambridge Institute for Medical Research (CIMR), University of Cambridge, Keith Peters Building, Biomedical Campus, Hills Rd, Cambridge CB2 0XY, United Kingdom; Cambridge Institute for Medical Research (CIMR), University of Cambridge, Keith Peters Building, Biomedical Campus, Hills Rd, Cambridge CB2 0XY, United Kingdom; Victor Phillip Dahdaleh Heart and Lung Research Institute, University of Cambridge, Papworth Road, Trumpington, Cambridge CB2 0BB, United Kingdom; Department of Medicine, University of Cambridge, Addenbrooke's Hospital (Box 157), Hills Road, Cambridge CB2 2QQ, United Kingdom; Victor Phillip Dahdaleh Heart and Lung Research Institute, University of Cambridge, Papworth Road, Trumpington, Cambridge CB2 0BB, United Kingdom; Department of Medicine, University of Cambridge, Addenbrooke's Hospital (Box 157), Hills Road, Cambridge CB2 2QQ, United Kingdom; Royal Papworth Hospital NHS Foundation Trust, Papworth Rd, Trumpington, Cambridge CB2 0AY, United Kingdom; Cambridge Institute for Medical Research (CIMR), University of Cambridge, Keith Peters Building, Biomedical Campus, Hills Rd, Cambridge CB2 0XY, United Kingdom; Department of Medicine, University of Cambridge, Addenbrooke's Hospital (Box 157), Hills Road, Cambridge CB2 2QQ, United Kingdom; Royal Papworth Hospital NHS Foundation Trust, Papworth Rd, Trumpington, Cambridge CB2 0AY, United Kingdom

**Keywords:** GCN2, EIF2AK4, pulmonary hypertension, PVOD, missense variants

## Abstract

Pulmonary arterial hypertension (PAH) is a disorder with a large genetic component. Biallelic mutations of *EIF2AK4*, which encodes the kinase GCN2, are causal in two ultra-rare subtypes of PAH, pulmonary veno-occlusive disease and pulmonary capillary haemangiomatosis. *EIF2AK4* variants of unknown significance have also been identified in patients with classical PAH, though their relationship to disease remains unclear. To provide patients with diagnostic information and enable family testing, the functional consequences of such rare variants must be determined, but existing computational methods are imperfect. We applied a suite of bioinformatic and experimental approaches to sixteen *EIF2AK4* variants that had been identified in patients. By experimentally testing the functional integrity of the integrated stress response (ISR) downstream of GCN2, we determined that existing computational tools have insufficient sensitivity to reliably predict impaired kinase function. We determined experimentally that several *EIF2AK4* variants identified in patients with classical PAH had preserved function and are therefore likely to be non-pathogenic. The dysfunctional variants of GCN2 that we identified could be subclassified into three groups: misfolded, kinase-dead, and hypomorphic. Intriguingly, members of the hypomorphic group were amenable to paradoxical activation by a type-1½ GCN2 kinase inhibitor. This experiment approach may aid in the clinical stratification of *EIF2AK4* variants and potentially identify hypomorophic alleles receptive to pharmacological activation.

## Introduction

Aberrant vascular remodelling in pulmonary arterial hypertension (PAH) raises pressures in the pulmonary vasculature to cause right heart failure [1]. Affected young adults often suffer progressive disease leading to premature death. Although classical PAH is most frequently caused by mutations in the TGFβ/BMP signalling axis [2–5], rare subtypes such as pulmonary veno-occlusive disease (PVOD) and pulmonary capillary haemangiomatosis (PCH) have distinct genetic associations and are refractory to current clinical management [6]. With no effective treatments apart from lung transplantation, death occurs within a year in 72% of patients diagnosed with these aggressive PAH subtypes [7].

**Figure 1:**
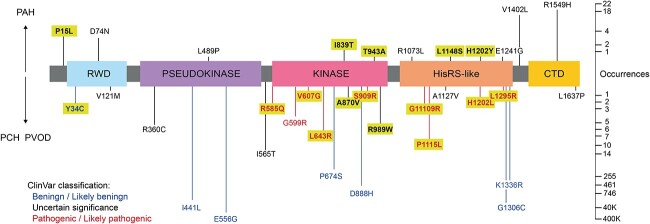
Schematic of known patient-specific missense variants. GCN2 schematic and its domains. Variants over the domain schematic were from patients with classical PAH. Variants below were from patients with PCH and PVOD. Variants reported to be benign/likely benign in ClinVar database*^33^* are in blue, those reported as pathogenic/likely pathogenic are in red, black represents variants of uncertain significance. Occurrences represent cumulative allele counts in the gnomAD database*^34^* or published reports^8–14^. Variants highlighted in yellow are analysed experimentally in this study.

Since the first report in 2014 linking biallelic mutations of *EIF2AK4* to PVOD [8], approximately one hundred *EIF2AK4* alleles have been reported to be associated with PAH and its subtypes [4, 8–14]. Although frameshift mutations constitute a large proportion, approximately a third [34] of these alleles are missense variants, the functional consequences of which are unknown (Fig. 1) [15]. Validating the pathogenicity of such variants of uncertain significance (VUSs) would aid in diagnosis, enable cascade genetic testing of relatives, and recruitment of patients to chemoprotective clinical trials [6].

*EIF2AK4* encodes GCN2, a large serine/threonine kinase homodimer that responds to amino acid depletion by monitoring the efficiency of protein synthesis through its interaction with stalled ribosomes [16, 17]. GCN2 is comprised of an N-terminal RWD domain (20–137) involved in protein–protein interactions, pseudokinase (276–539) and kinase domains (585–1016) including GCN2’s dimerisation interface, an HisRS-like domain (1058–1490) and C-terminal domain (1533–1649) where binding to ribosomes and recognition of uncharged tRNAs occur [18]. When activated, GCN2 triggers a cellular signalling pathway termed the integrated stress response (ISR) by phosphorylating the α subunit of eukaryotic translation initiation factor 2 (eIF2α) [19, 20]. This attenuates most protein synthesis, while enhancing the translation of ISR-specific mRNAs owing to the presence of upstream open reading frames (uORFs) in their 5’UTRs [21]; an example is the transcription factor ATF4. PPP1R15A, a selective eIF2α phosphatase subunit, is similarly regulated and its expression eventually terminates the ISR [20–22]. Due to its fundamental biological roles, disruption of the ISR is implicated in many diseases [23–25].

Technological advances have made genomic sequencing readily available in the clinic, leading to a proliferation in the number of VUSs encountered. Currently, strategies to predict the impact of novel genetic variants are largely restricted to computational methods that rely on evolutionary conservation (e.g. SIFT, PolyPhen) [26] or integrate a range of scores from existing predictive tools (e.g. CADD, REVEL) [27, 28]. Other methodologies include the computation of folding free energy differences (FoldX) [29], which predicts protein stability. More recently, deep learning approaches have been developed (EVE, AlphaMissense) [30, 31] that account for both evolutionary constraints and structural information. Although these have improved predictive accuracy, computational approaches remain imperfect. We set out to examine patient-specific *EIF2AK4* missense mutations both *in silico* and experimentally using existing bioinformatic tools and cell biological assays. In so doing, we subclassified PAH-associated GCN2 variants in functional (likely benign), destabilised/misfolded, or kinase impaired. Interestingly, a subset of the kinase impaired variants showed preserved target engagement. These hypomorphic variants were amenable to pharmacological rescue using an ATP-competitive GCN2 inhibitor [32]. Based on these results, we propose a simple methodology for the experimental validation of the functionality of *EIF2AK4* VUSs, which outperforms existing computational approaches.

## Results

### Computational analysis of PAH-associated *EIF2AK4* missense variants suggests heterogeneity

Thirty-four missense variants of *EIF2AK4* (RefSeq: NC_000015.10) have been published [8–14] or reported in ClinVar [33] to be associated with PAH (Table 1). Genome sequencing of the general population [34] suggests that most of these variants are rare, with allele frequencies <  0.01, though three, I441L, E556G and G1306C, are common polymorphisms with frequencies > 0.1 (Fig. 1). Nine have already been classified as pathogenic/likely pathogenic (R585Q, G599R, V607G, L643R, S909R, G1109R, P1115L, H1202L and L1295R, depicted in red) and seven as benign/likely benign, including the three common variants (Y34C, I441L, E556G, P674S, D888H, G1306C and K1336R, depicted in blue), but most remain uncharacterised VUSs (Fig. 1, Table 1).

**Table 1:** Known pulmonary hypertension-associated missense variants of GCN2.

DNA Base	Amino Acid	Pubication	ClinVar classification	Patient Diagnosis	SIFT	PolyPhen2	CADDv1.6	REVEL	Alpha Missense	FoldX: ΔΔG (kcal/mol)	Integrative *in silico* functional prediction
44C > T	**P15L**	Hadinnapola 2017	VUS	PAH (Het)	T	0	22.6	0.133	0.0834	0.23	Benign
145-2A > G	**Y34C**	UK cohort (unpublished)	Benign/likely-benign	PVOD (C Het)	T	0.999	32	0.634	0.4589	3.79	Misfolded
220G > A	**D74N**	Hadinnapola 2017	VUS	PAH (Het)	NT	0.954	28	0.166	0.1726	0.38	Benign
361G > A	**V121M**	Montani 2017	VUS	PVOD (C Het)	T	1	33	0.467	0.7125	−1.04	Benign
1078C > T	**R360C**	ClinVar RCV001287133	VUS	PCH	NT	0.999	26.3	0.566	0.083	1.68	Uncertain
1321A > C	**I441L**	ClinVar RCV000999785	Benign/likely-benign	PCH	T	0.001	16.9	0.075	0.0839	−0.70	Benign
1466C > T	**L489P**	Gómez 2015	VUS	PAH	NT	1	29	0.895	0.9737	7.89	Misfolded
1667A > G	**E556G**	ClinVar RCV000755517	Benign/likely-benign	PCH	T	0	22.5	0.088	0.0523	0.63	Benign
1694 T > C	**I565T**	ClinVar RCV001285279	VUS	PCH	T	0.835	22.2	0.307	0.2246	−0.36	Benign
1754G > A	**R585Q**	Eyries 2014; Montani 2017	Pathogenic/likely-pathogenic	PVOD; PCH	NT	1	32	0.861	0.9272	2.12	Kinase-deficient
1795G > C	**G599R**	Hadinnapola 2017	Pathogenic/likely-pathogenic	PAH; PCH (Hom)	NT	1	32	0.952	0.9991	0.83	Kinase-deficient
1820 T > G	**V607G**	Hadinnapola 2017	Pathogenic/likely-pathogenic	PAH (C Het); PCH	NT	0.998	33	0.557	0.8039	4.73	Misfolded
1928 T > G	**L643R**	Eyries 2014; Levy 2016; Montani 2017	Pathogenic/likely-pathogenic	PVOD; PCH	NT	0.999	32	0.889	0.9911	9.65	Misfolded
2020C > T	**P674S**	ClinVar RCV001286399	Benign/likely-benign	PCH	T	0.029	12.1	0.077	0.0694	−1.45	Benign
2516 T > C	**I839T**	Hadinnapola 2017	VUS	PAH (Het)	NT	0.99	29.6	0.781	0.9854	2.90	Benign
2609C > T	**A870V**	Montani 2017	VUS	PVOD (C Het)	T	1	28.1	0.658	0.9943	0.95	Uncertain
2662G > C	**D888H**	ClinVar RCV001287878	Benign/likely-benign	PCH	NT	0.998	24.8	0.237	0.1005	0.59	Benign
2727C > G	**S909R**	Hadinnapola 2017	Pathogenic/likely-pathogenic	PAH (Het); PCH	NT	1	28	0.446	0.9985	5.67	Misfolded
2827A > G	**T943A**	Hadinnapola 2017	VUS	PAH (C Het)	NT	0.997	25.4	0.464	0.8597	1.54	Benign
2965C > T	**R989W**	Eichstaedt 2022	VUS	PVOD	NT	1	28.9	0.862	0.9838	38.59	Misfolded
3218G > T	**R1073L**	Hadinnapola 2017	VUS	PAH	NT	0.995	32	0.466	0.3374	0.15	Benign
3325G > A	**G1109R**	Hadinnapola 2017	Pathogenic/likely-pathogenic	PAH (C Het); PCH	NT	1	32	0.766	0.9856	9.20	Uncertain
3344C > T	**P1115L**	Gómez 2015; Tenorio 2015	Pathogenic/likely-pathogenic	PAH; PVOD; IPAH; PCH	NT	1	29.7	0.702	0.8415	1.46	Uncertain
3380C > T	**A1127V**	Eichstaedt 2022	VUS	PVOD	T	1	32	0.262	0.6049	6.08	Misfolded
3443 T > C	**L1148S**	Eichstaedt 2022	VUS	CTD-APAH	T	1	28.4	0.341	0.2648	1.11	Benign
3604C > T	**H1202Y**	Hadinnapola 2017	VUS	PAH (Het)	T	0.996	26.5	0.716	0.812	−0.44	Benign
3605A > T	**H1202L**	Hadinnapola 2017	Pathogenic/likely-pathogenic	PAH; PCH (Hom)	NT	0.998	28.4	0.802	0.9507	3.00	Uncertain
3722A > G	**E1241G**	Hadinnapola 2017	VUS	PAH	NT	0.971	33	0.342	0.7069	1.50	Benign
3884 T > G	**L1295R**	Hadinnapola 2017	Pathogenic/likely-pathogenic	PAH; PCH (C Het)	NT	1	28.8	0.814	0.9408	4.89	Misfolded
3916G > T	**G1306C**	ClinVar RCV000999798	Benign/likely-benign	PCH	NT	1	31	0.307	0.2596	2.26	Uncertain
4007A > G	**K1336R**	ClinVar RCV003103836	Benign/likely-benign	PCH	T	0	15.8	0.063	0.0564	0.14	Benign
4204G > T	**V1402L**	Montani 2017	VUS	IPAH	T	0.997	24.7	0.285	0.4847	−0.19	Benign
4646G > A	**R1549H**	Hadinnapola 2017	VUS	PAH (Het)	NT	0.998	32	0.318	0.6517	0.30	Benign
4910 T > C	**L1637P**	Montani 2017	VUS	PVOD (C Het)	NT	1	31	0.624	0.9982	5.98	Misfolded
	**K619R**		-	kinase-dead	NT	1	32	0.783	0.9769	0.20	Kinase-deficient

ClinVar classification (VUS: variant of uncertain significance) and patient diagnosis where available: Hom = homozygous, Het = heterozygous, C Het = compound heterozygous. Scores SIFT (T: tolerated; NT: not tolerated), PolyPhen2, CADD v1.6, REVEL and AlphaMissense (high scores corresponding to more severe consequences). FoldX used to estimate difference in Gibbs free energy (ΔΔG, kcal/mol) between a variant and the non-mutated GCN2 (scores > 1 indicate predicted destabilisation, scores > 3 indicate predicted severe misfolding).

We applied existing computational methods including SIFT, PolyPhen2, CADDv1.6, REVEL and AlphaMissense in an effort to predict the functional significance of these GCN2 variants [26–28, 30] (Table 1). Higher scores represent higher predicted severity. We also used FoldX5.0 to estimate differences in Gibbs free energy (ΔΔG) between wildtype GCN2 and each variant (Table 1) [29]. Although broadly concordat, the algorithms yielded several discordant results. For example, although H1202Y was predicted to maintain stable folding by FoldX, and tolerated by SIFT and CADD, it was categorised as severe/likely pathogenic by the other methods. Conversely, Y34C was predicted to be destabilised by FoldX (ΔΔG > 1.5 kcal/mol) and likely-pathogenic by PolyPhen, CADD and REVEL, but tolerated by SIFT and categorised as ambiguous by AlphaMissense. Nevertheless, amid a few uncertain results, by integrating these computational methods we predicted missense variants of GCN2 to be either (i) benign, (ii) misfolded, or (iii) kinase deficient (Summarised in Table 1).

### Expression of GCN2 variants and ISR reporter activation

**Figure 2:**
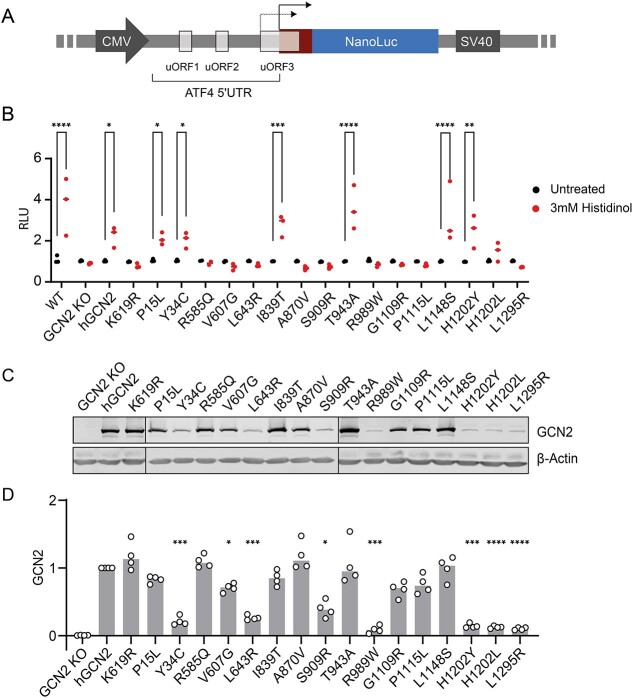
Variant in cell activity and expression. (A) ISR reporter construct comprising 5’ UTR of human ATF4 containing upstream open reading frames (uORF) cloned in frame with NanoLuc® luciferase. CMV promoter and SV40 enhancer. Under conditions of low eIF2α phosphorylation, uORF3 is translated resulting in low reporter signal. When eIF2α phosphorylation increases, ATF4 initiator AUG is translated leading to NanoLuc reporter synthesis. (B) Reporter activity (relative light units, RLU) in reporter HeLa cells without and with 3 mM histidinol 6 h. WT cells with endogenous GCN2. GCN2 KO = knockout cells lacking functional GCN2. Variants expressed in the GCN2 KO lines are indicated. K619R = kinase dead negative control. n = 3 biological replicates; 2-way ANOVA with group comparisons. (C) Representative immunoblots of GCN2 variant expression. Note—For clarity two blots are joined as indicated by vertical black lines between lanes 2&3, 3&4, and 11&12. (D) Quantification of C. n = 4, 1-way ANOVA with multiple comparisons. ^*^P < 0.05, ^*^^*^P < 0.01, ^*^^*^^*^P < 0.001, ^*^^*^^*^^*^P < 0.0001.

To test the *in silico* predictions, we next performed experimental validation in cultured cells. A bioluminescent ISR reporter was generated by fusing the 5’UTR of ATF4 with NanoLuc® luciferase (ATF4::NanoLuc, Fig. 2A). We obtained optimal results when using human rather than murine ATF4, driven by a CMV promoter/SV40 enhancer (data not shown). uORFs in the 5’UTR of ATF4 mRNA impose translational regulation on the downstream coding sequence [21]. When endogenous GCN2 was deleted in reporter HeLa cells (GCN2 KO, Fig. S1), as expected activation of the ISR reporter by the histidyl-tRNA synthetase inhibitor histidinol was ablated (Fig. 2B). Re-expression of wild type human GCN2, but not a kinase-dead control mutant (K619R) rescued ATF4::NanoLuc responsiveness to histidinol, validating the system (Fig. 2B).

Sixteen GCN2 exemplar variants were selected as representative of each class of functional prediction, across a range of disease severities and distributed throughout the GCN2 protein (highlighted in Fig. 1). Each variant was expressed in the GCN2-deleted reporter cells and bioluminescence was measured without or with histidinol treatment (Fig. 2B). We noted a striking correlation between ATF4::NanoLuc reporter activation and diagnosis (Table 2). In all but one case, when reporter activation was preserved (P15L, I839T, T943A, L1148S, H1202Y), the clinical diagnosis had been of classical PAH, rather than either PVOD or PCH. The exception was Y34C, which had preserved reporter activation despite having been identified in an individual with PVOD (that patient also had a high impact variant in their other *EIF2AK4* allele: Lys190GlufsTer8, c.567dup). All other variants identified in PVOD or PCH showed impaired ATF4::NanoLuc responsiveness to histidinol (Fig. 2B).

**Table 2:** Variants analysis summary.

Base	Amino Acid	Diagnosis	ATF4 Induction	Expression Level	GCN2-T899 autophosphorylation	Dimerisation	eIF2α-S51 phosphorylation	Classification
44C > T	**P15L**	PAH (Het)	Yes	0.83	Yes	-	-	Benign
145-2A > G	**Y34C**	PVOD (C het)	Yes	0.21	Yes	-	-	Destabilised
1754G > A	**R585Q**	PVOD; PCH	No	1.09	Yes	Yes	Yes (32.6%)	Hypomorphic
1820 T > G	**V607G**	PAH; PCH	No	0.71	Yes	Yes	Yes (42.8%)	Hypomorphic
1928 T > G	**L643R**	PVOD; PCH	No	0.26	No	Yes	No	Kinase-dead
2516 T > C	**I839T**	PAH (Het)	Yes	0.85	Yes	-	-	Benign
2609C > T	**A870V**	PVOD (C het)	No	1.00	No	-	-	Kinase-dead
2727C > G	**S909R**	PAH; PCH	No	0.39	No	Yes	-	Kinase-dead
2827A > G	**T943A**	PAH	Yes	1.06	Yes	-	-	Benign
2965C > T	**R989W**	PVOD	No	0.09	No	-	-	Misfolded
3325G > A	**G1109R**	PAH; PCH	No	0.68	Yes	-	Yes (27.2%)	Hypomorphic
3344C > T	**P1115L**	PAH; PVOD; IPAH; PCH	No	0.76	Yes	-	Yes (12.9%)	Hypomorphic
3443 T > C	**L1148S**	CTD-APAH	Yes	0.99	Yes	-	-	Benign
3604C > T	**H1202Y**	PAH	Yes	0.14	Yes	-	-	Benign
3605A > T	**H1202L**	PAH; PCH (Hom)	No	0.12	Yes	-	No	Misfolded
3884 T > G	**L1295R**	PAH; PCH (C Het)	No	0.10	No	-	No	Misfolded
	**K619R**	kinase-dead	No	1.17	No	-	No	Kinase-dead

**Figure 3:**
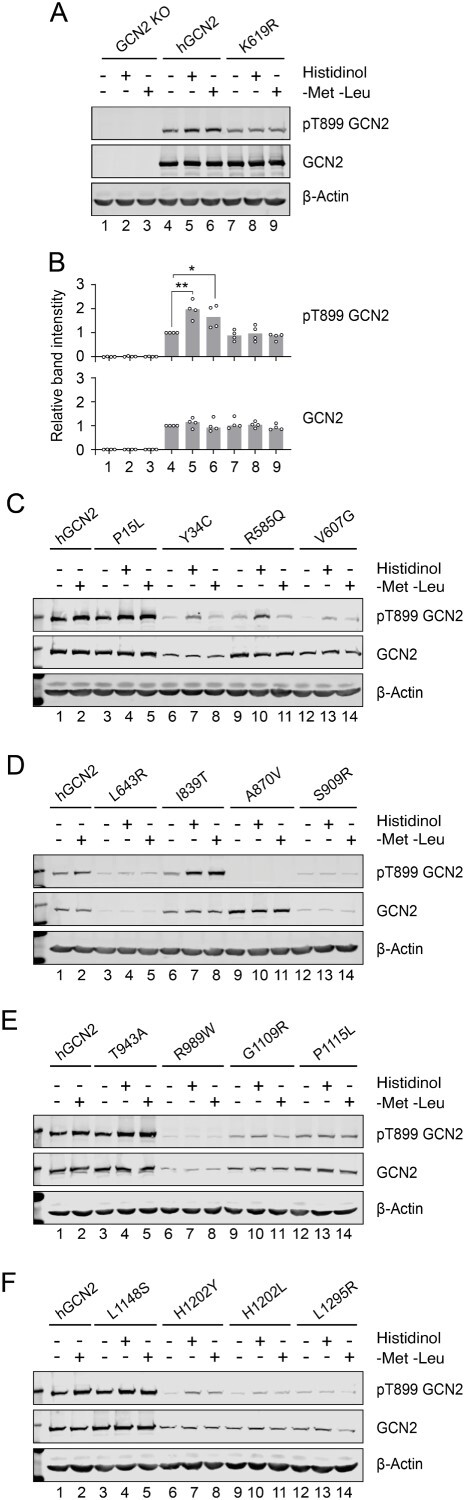
Stress-dependent activation of GCN2. (A) Representative immunoblot of GCN2 KO HeLa cells transiently transfected with constructs encoding full-length human GCN2 (hGCN2) or kinase-dead negative control (K619R). Hygromycin-selected pools were treated with either 7 mM histidinol or starved of methionine and leucine (−met -Leu) for 7 h. (B). Quantifications of a. n = 4 biological replicates normalised to untreated, hGCN2-transfected (lane 4). 1-way ANOVA with multiple comparisons (meaningful comparisons are shown). ^*^P < 0.05, ^*^^*^P < 0.01. (C-F) representative immunoblots in GCN2 KO HeLa cells transiently transfected with GCN2 variants treat as in a. n = 3 biological replicates.

ATF4 induction and protein expression from Fig. 2. T899 autophosphorylation from Fig. 3. Dimerisation from Fig. 5. In vitro eIF2α phosphorylation from Fig. 6.

Next, protein expression of the sixteen GCN2 variants was determined by immunoblotting (Fig. 2C-D). Significantly reduced expression relative to wildtype GCN2 was observed for eight variants (Y34C, V607G, L643R, S909R, R989W, H1202Y, H1202L, L1295R). Of these, despite their low expression, two retained significant ATF4::NanoLuc reporter activation (Y34C and H1202Y). Conversely, three of these naturally occurring variants lacked reporter activity despite preserved expression (R585Q, G1109R and P1115L), similar to that seen for the artificial kinase-dead control (K619R). Of note, V607G showed no reporter activation despite only modestly reduced expression. Expression levels of GCN2 variants therefore do not fully reflect activity.

### GCN2 autophosphorylation is necessary but not sufficient for ISR induction

Autophosphorylation of GCN2 at T899 correlates with kinase activation in most circumstances [32, 35]. Treatment with histidinol or starvation of amino acids, a physiological stimulus of the kinase, increased T899 phosphorylation of wildtype but not kinase-dead K619R GCN2 expressed in knockout cells (Fig. 3A-B & S1B-C). When the naturally occurring variants were tested, eleven were capable of T899 autophosphorylation, while five were not (L643R, A870V, S909R, R989W, L1295R) (Fig. 3C-F, summarised in Table 2). These results show that stressful stimuli triggered T899 autophosphorylation in all GCN2 variants capable of activating the ATF4::NanoLuc reporter, but also in five ISR-deficient variants (R585Q, V607G, G1109R, P1115L, H1202L) albeit only weakly (Figs. 3C-F, summarised in Table 2).

**Figure 4:**
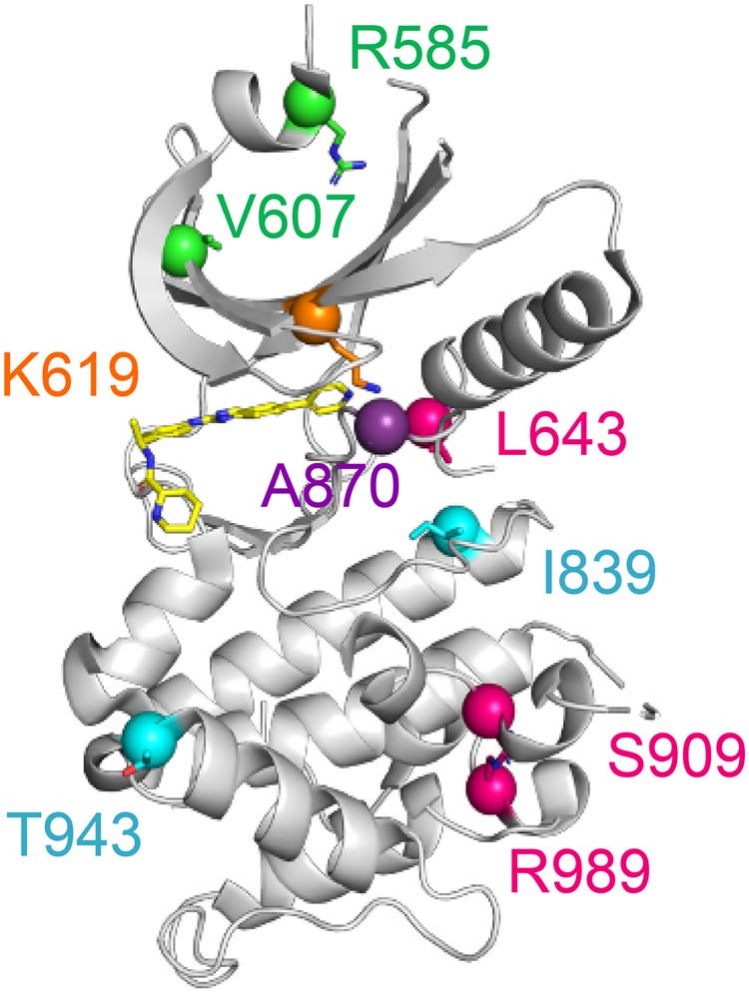
Structure of GCN2 kinase domain with mapped residues mutated in patients. Human GCN2 kinase domain structure (from PDB accession: 7QWK)^41^ highlighting the location of residues mutated in PAH, as well as K619 (in orange) mutated in the kinase-dead control. Mutations that led to the loss of kinase activity include A870V, located in the activation loop (in purple), L643R in the hinge region (in magenta), S909R and R989W which are in the core of the C-lobe (in magenta). Variants I839T and T943A (in cyan), localised at the edge of the kinase helix without introducing bigger sidechains, did not affect folding and retained kinase activity. Variants R585Q and V607G localised in the N-lobe (in green) were hypomorphic. Bound ATP is shown in yellow.

These results suggested that GCN2 autophosphorylation at T899 is necessary but not sufficient for ISR induction. From a structural perspective, the A870 (Fig. 4, in purple) is located on the kinase activation loop, in close proximity to residue K619 (in orange) of the N-lobe, essential for kinase activity (K619R yields a dead kinase). Mutations in these key residues led to total loss of autophosphorylation and kinase activity. L643, S909 and R989 (Fig. 4, in magenta) are essential residues for the tight packing of the kinase domain C-lobe. L643R, S909R, R989W introduce larger and differently charged sidechains into the hinge region or the C-lobe of the kinase, disrupting the local protein structure required for kinase activity. On the other hand, I839 and T943 (Fig. 4, in blue) are located at the edge of the kinase helix. The I839T and T943A variants do not introduce larger sidechains nor disrupt local packing, hence retaining activity.

**Figure 5:**
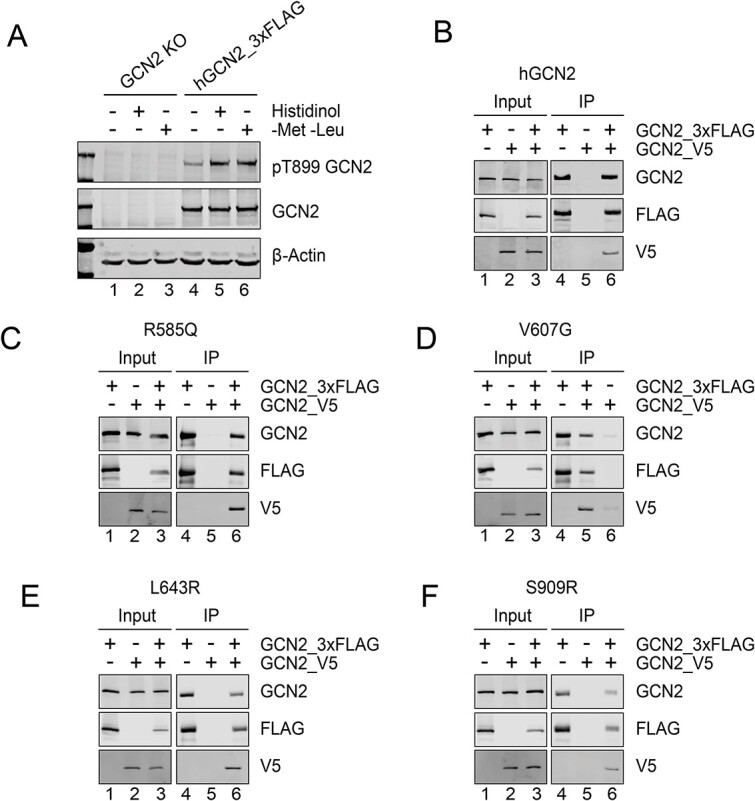
Dimerisation of variants of GCN2 variants. (A) Representative immunoblot of GCN2 KO HeLa cells transiently transfected with construct encoding 3xFlag tagged human GCN2 (hGCN2_3xFLAG) treated with either 7 mM histidinol or starved of methionine and leucine (−met -Leu) for 7 h. n = 3 biological replicates (B-F) anti-FLAG immunoprecipitation-immunoblots from GCN2 KO cells transiently transfected with constructs encoding GCN2 variants tagged at the C-terminus with either 3xFLAG (GCN2_3xFLAG) or V5 (GCN2_V5). Lysates (lanes 1–3) and immunoprecipitates (lanes 4–6). Note co-immunoprecipitation of each pair of constructs consistent with dimer formation.

Inactive GCN2 exists as an antiparallel homodimer and transitions to a parallel conformation on activation [36, 37]. In yeast, stabilisation of the active state depends on the establishment of an intramolecular salt-bridge in the active conformation between residues R594 and D598, corresponding to R585 and E589 in the human protein [38] (Fig. S2). The patient-derived R585Q and V607G variants, localised in the kinase domain N-lobe (Fig. 4, in green) were unable to activate the ATF4::NanoLuc reporter despite preserved expression (Fig. 2) and autophosphorylation (Fig. 3). Since the R585Q substitution is predicted to reposition the dimerisation interface salt-bridge required for activation, we sought to test if dimerisation was impaired. GCN2 constructs were generated tagged at the C-terminus with either 3xFlag or V5. Tagging did not impair GCN2 activity (Fig. 5A). When co-expressed in GCN2 deleted cells, wildtype GCN2-3xFlag and GCN2-V5 formed mixed dimers detectable by anti-FLAG co-immunoprecipitation (Fig. 5B). The R585Q variant similarly formed mixed dimers that could be co-immunoprecipitated (Fig. 5C). The ISR-deficient kinase domain variants V607G, L643R, and S909R, predicted *in silico* to affect the folding of the kinase domain (Table 1), were similarly able to form mixed dimers, suggesting that, at least for these variants, loss of dimerisation does not contribute to their impaired function (Fig. 5C-F).

### Hypomorphic GCN2 variants can be activated by an ATP-competitive inhibitor

**Figure 6:**
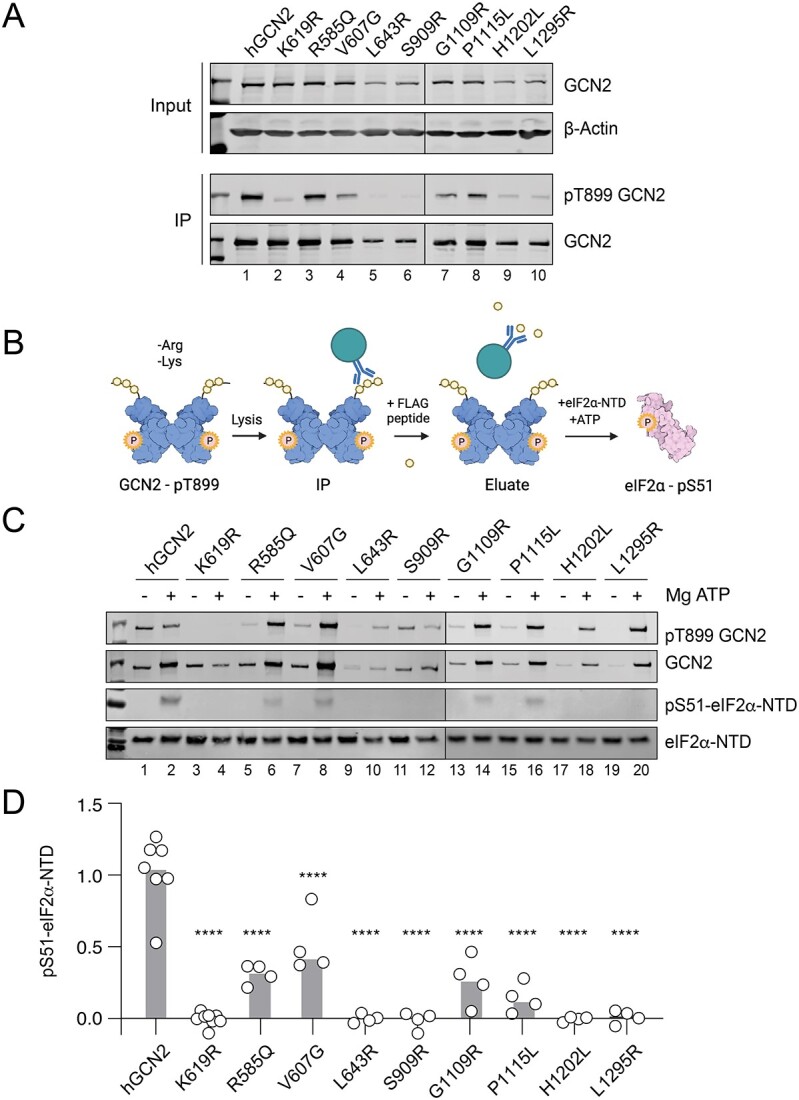
*In vitro* GCN2 kinase assay. (A) Representative immunoprecipitation-immunoblots from cells expressing 3xFlag tagged GCN2. Cells were starved of arginine and lysine for 6 h before harvesting. Intact GCN2 was eluted by competition via addition of excess FLAG peptide. Phosphorylation on threonine 899 reports autophosphorylation, note its absence for the K619R kinase-dead control. Note—For clarity two blots are joined as indicated by vertical black lines between 6&7. (B) Schematic of *in vitro* kinase assay drawn using Biorender.com. Tagged protein immunopurified using anti-FLAG beads then eluted with FLAG peptide. Bacterially expressed recombinant eIF2α-N-terminal domain (NTD) served as a specific substrate. (C). Representative immunoblots of reaction products. Note—For clarity two blots are joined as indicated by vertical black lines between lanes 12&13. (D) Quantification of C; data presented with median value. n = 4; 1-way ANOVA, compared to hGCN2-transfected control. ^*^^*^^*^^*^P < 0.0001.

We then sought to test whether autophosphorylation-competent, but ISR-deficient variants might have lost the ability to engage the substrate eIF2α. Tagged GCN2 was recovered by immunoprecipitation of 3xFlag from lysates of cells starved of amino acids (Fig. 6A). Immunopurified kinases were then tested for their ability to phosphorylate the N-terminal domain of eIF2α (eIF2α-NTD) *in vitro* (Fig. 6B-D). Wildtype but not kinase-dead K619R GCN2 showed enhanced T899 autophosphorylation when incubated with Mg-ATP, leading to increased phospho-GCN2 immunoreactivity and slower migration on SDS-PAGE (Fig. 6B-D). When incubated with Mg-ATP and eIF2α-NTD, wildtype but not kinase dead K619R GCN2 phosphorylated eIF2α-NTD on serine 51 (Fig. 6C-D). The autophosphorylation-competent variants R585Q, V607G, G1109R and P1115L, but not the kinase-dead S909R (Figs. 3C,E and 6B) also autophosphorylated their activation loop at T899 when incubated with Mg-ATP *in vitro*, and phosphorylated eIF2α-NTD albeit only weakly (Fig. 6C-E). The variants L643R, S909R, H1202L and L1295R, which showed either weak or no autophosphorylation combined with low expression (Fig. 6A,C) failed to phosphorylate eIF2α-NTD (Fig. 6C-D). These data suggest that while some PVOD/PCH-associated variants preserve target engagement *in vitro*, their weak kinase activity appears insufficient for downstream signalling in cells. We classified these variants as hypomorphic.

**Figure 7:**
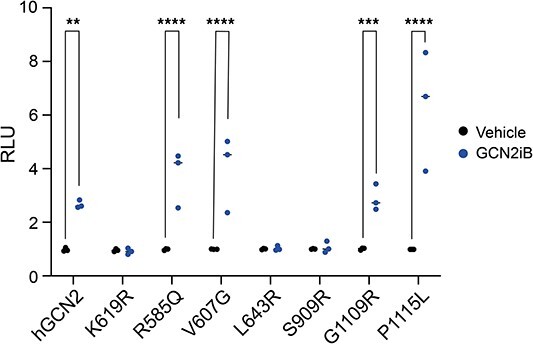
Activity of hypomorphic variants of GCN2 treated with Gcn2iB. ATF4::nanoLuc ISR reporter activity in GCN2 KO cells transiently transfected with constructions wild-type (hGCN2), kinase-dead (K619R), or selected patient variants. Cells were treated with 25 nM Gcn2iB for 6 h before luciferase assay. n = 3 biological replicates; 2-way ANOVA with group comparisons. ^*^P < 0.05, ^*^^*^P < 0.01, ^*^^*^^*^P < 0.001, ^*^^*^^*^^*^P < 0.0001.

It was recently shown that GCN2 can be activated paradoxically by sub-inhibitory concentrations of ATP-competitive kinase inhibitors [39, 40]. Carlson *et al.* showed activity of GCN2-R585Q, one of the hypomorphic variants identified here, though not the L643R variant, by treatment with the type-1½ kinase inhibitor Gcn2iB [32]. We therefore tested the ability of such a small molecule, to activate the ATF4::NanoLuc reporter in cells expressing PAH-associated GCN2 variants. We found that all the identified hypomorphs (R585Q, V607G, G1109R and P1115L) were rescued in their reporter activation by Gcn2iB, while misfolded or kinase-dead variants were not (K619R, L643R and S909R; Fig. 7).

## Discussion

Detection of biallelic pathogenic *EIF2AK4* mutations establishes the diagnosis of PVOD or PCH without the need for histological confirmation [6]. Validating the pathogenicity of *EIF2AK4* variants is therefore of significant diagnostic value, which is important since therapies developed for classical PAH can be detrimental in these rare subtypes [7]. Segregation studies, although the gold standard, are not always feasible. By contrast, genetic testing is routinely used in clinical practice. We found that integrative computational analysis failed to identify some ISR-defective variants of GCN2. However, our *in cellulo* assay using a sensitive ATF4::nanoLuc reporter cell line could reliably identify pathogenic variants of GCN2. This approach is simple and inexpensive, making it a feasible addition to characterisation workflows in specialist clinical practice.

We showcased this approach by evaluating sixteen of the thirty-four known missense variants of GCN2 associated with PAH. Our observation that variants identified by genomic sequencing of individuals with classical PAH had preserved ISR reporter activity is consistent with GCN2 playing only a minor, or even no, role in that condition. Conversely, variants identified in individuals with either PVOD or PCH showed loss of ISR functionality, underlining the key role played by GCN2 in these disorders. The Y34C variant was a notable exception, maintaining some ISR reporter activity despite having been identified in an individual with PVOD. Of note, that patient also harboured a high-impact mutation of their second *EIF2AK4* allele. In our study, GCN2 Y34C was expressed at a significantly reduced level compared to the wild-type protein, raising the possibility that when combined with a null allele, the level of GCN2 generated might be insufficient to prevent development of the disease.

Importantly, we identified a subset of GCN2 variants with preserved target engagement but reduced kinase activity. R585Q and V607G locate to the kinase domain N-lobe which is dominated by an anti-parallel beta-sheet and contains most of the residues involved in ATP binding. This portion of the kinase also participates in dimerisation and stabilisation of the active parallel conformation [41]. Though our data exclude lack of dimerisation, it remains possible that mutants in the N-lobe could affect dimer activation. Conversely, G1109 and P1115, corresponding to G1085 and Q1091 in yeast, have been shown to be involved in tRNA binding [37]. G1109R and P1115L mutations change mainchain flexibility and introduce bulky sidechains, likely to alter such interaction. Strikingly, these hypomorphs could be activated by the ATP-competitive inhibitor Gcn2iB, a type 1½ inhibitor that stabilises the enzyme in a non-productive, yet active-like conformation (DFG-in, αC helix-out). It is believed that such binding to one protomer of a GCN2 dimer causes activation of the second drug-free promoter [39]. This suggests a potential therapeutic strategy for individuals with such hypomorphic variants. Hypomorphs can be identified by our ATF4::nanoLuc reporter assay by treatment with Gcn2iB.

The experimental validation of GCN2 variants enabled us to evaluate computational predictive methods. It is recognised that evolutionary-based methods relying on homologous sequence alignment (e.g. SIFT, MAPP, PANTHER) are outperformed as stand-alone tools by approaches that integrate additional information such as structural features [42]. We compared the integrative tools PolyPhen2 and CADD and found that out of sixteen variants they incorrectly predicted enzyme activity in 5 and 6 instances respectively, giving a positive predictive value of approximately only 60%. Indeed, functional predictions using these methods are not recommended for diagnostic purposes for this reason [43]. Accuracy was improved with machine learning-based approaches, but even these rarely exceed 80% accuracy [44]. It has been estimated that 75% of disease-causing variants are linked to protein destabilisation [45]. The computation of Gibbs free energy variations with FoldX was recently reported as the best performing method for the identification of disease-causing mutations [46]. FoldX predicted protein expression levels of GCN2 missense variants in our cell system, but the existence of stable but kinase-dead or hypomorphic variants limits its value. Nevertheless, integrating conservation-based approaches with structural features improves predictive performance. Meta-predictors, such as the ensemble method REVEL [28], are better correlated with benchmark clinical datasets like ClinVar (reviewed in reference (47)). Although better than other tools, REVEL only returned 75% accuracy in our study. Recently AlphaMissense was developed, a deep-learning AlphaFold-derived system that combines residue structural context with unsupervised modelling of evolutionary constraints by comparing related sequences, claiming 90% accuracy in predicting the pathogenicity of missense variants when tested against the ClinVar dataset [31]. When using ISR-reporter activation as our gold standard, AlphaMissense incorrectly assigned 3 of the 16 GCN2 variants examined. The complexity of modelling protein–protein and protein-ribosome interactions adds to the challenge of using computational approaches to classify GCN2 variants [16, 35].

In summary, *in cellulo* evaluation of ISR signalling using an ATF4::Nanoluc reporter outperformed existing computational approaches. This approach can not only identify pathogenic variants but can also recognise hypomorphs that can be revitalised by an ATP-pocket-binding small molecule drug.

## Materials and methods

### Cloning and plasmids

All cloning and mutagenesis were performed via Gibson Assembly. A human codon-optimised GCN2 ORF was cloned into pcDNA3.1-Hygro(+) vectors and tagged with either 3xFLAG or V5 at the C-terminus. Site-directed mutagenesis was performed with specific primers for 16 naturally occurring variants and a kinase-dead control. A human ATF4 5’UTR ORF was inserted into pGL4.2 vectors and cloned in frame with Nluc-PEST® luciferase. A stop codon was inserted before the C-terminal degron to allow accumulation of the reporter.

### CRISPR/Cas9 knockout of *EIF2AK4* in HeLa cells

Human *EIF2AK4* specific guide RNAs were selected from the Brunello sgRNA library [48]. After primer duplex formation, guides were inserted in pSpCas9(BB)-2A-mCherry plasmids. Parental HeLa cells, cultured in DMEM supplemented with 10% FBS were transfected in 6-well dishes using 1 μg DNA and Lipofectamine 2000 (1:3 ratio) in OptiMEM for 24 h. At day 3 post-transfection mCherry-positive cells were sorted on a DB Melody cell sorter, as single cells into 96 well plates. Clones were screened by GCN2-targeting western blotting. Genetic mutations in KO clones were confirmed by genomic DNA extraction (100 mM Tris, 5 mM EDTA, 200 mM NaCl, 0.25% SDS, 0.2 mg/ml Proteinase K: incubation at 50°C overnight, then for 20 minutes at 98°C and clarification in a benchtop centrifuge at 10 000 g for 5 minutes), PCR to amplify the locus targeted by the guide and subsequent NGS. Data were analysed using MacVector.

### Transfection and cell treatments

2×10^5^ HeLa cells were plated in 6-well dishes and let attach overnight before transfection. 1 μg of plasmid DNA was mixed with Lipofectamine 2000 (1:3 ratio) in OptiMEM and incubated for 20 minutes at room temperature. 500 μL of transfection medium were added onto the washed cells and topped up with additional 500 μL of 10% FBS-DMEM. Transfection medium was removed after 24 h. On day 2 cells were split and transfected cells were selected by 300 μg/ml hygromycin treatment for 3 days. Cells were then maintained in 150 μg/ml hygromycin 10% FBS-DMEM. Cell treatments were carried out as follows: 7 mM histidinol for 7 h (for western blotting); amino acid starvation in SILAC medium supplemented with 10% dialysed FBS and 25 mM D-glucose (after 1× wash in PBS) without leucine only, leucine and methionine or lysine and arginine, for 7 h.

### Immunoblotting

Cells were washed with PBS on ice and lysed in low-salt buffer (Buffer H: 10 mM HEPES pH 7.9, 50 mM NaCl, 0.5 M sucrose, 0.1 mM EDTA, 0.5% TX-100, 1 mM DTT, 10ul/ml cOmplete™ Protease Inhibitor Cocktail (Roche, SKU 11836170001), phosphatase inhibitor cocktail mix (10 mM tetrasodium pyrophosphate, 17.5 mM beta glycerophosphate, 100 mM sodium fluoride), 1 mM PMSF) and 10 mM DTT. Samples were clarified at 4°C in a benchtop centrifuge at 10′000 g for 10 minutes. Protein concentrations were estimated using Pierce BCA assay following the manufacturer’s instructions and equalised in lysis buffer and 6X loading buffer (1X: 60 mM Tris–HCl pH 6.8, 10% glycerol, 2% SDS, 0.02% bromophenol blue, 1 mM DTT). 8–14% polyacrylamide, 0.4% SDS gels were casted for SDS-PAGE. 60-80 μg of protein were loaded per lane. Proteins were transferred to 25 μm nitrocellulose membranes using 5% methanol transfer buffer. Blocking was done in 5% BSA in TBS as well as incubations with antibodies. Antibodies: GCN2 (in house, Ron lab: NY168); GCN2-phosphorT899 (Abcam, ab75836); β-actin (Abcam, ab8226); FLAG (Invitrogen, 740 001); V5 (Abcam, ab27671).

### NanoGlo® luciferase assay

GCN2 KO, ATF4::NanoLuc reporter cells were transfected with GCN2 variant constructs as described above. Cells were seeded in 384-well plates at a density of 2.5×10^3^ cells per well in 20 μL. Left-over cells were pelleted and lysed to confirm transfection efficiency via western blot. Wells were previously filled with 5 μL of 15 mM histidinol (5X) in 10% FBS-DMEM (final concentration 3 mM), or 125 nM (5X) Gcn2iB (final concentration 25 nM). After 6 h of incubation at 37°C and 5% CO_2_, 25 μL of NanoGlo® luciferase assay reagent (buffer + substrate, 50:1) were added to the wells. Plates were mixed with orbital shaking for 1 minute and after 10 minutes of incubation at room temperature, bioluminescence signal (360-545 nm) was acquired on a Tecan Spark plate reader.

### Dimerisation assay

GCN2 KO HeLa cells were transfected with 3xFLAG and/or V5 tagged GCN2 variant constructs as described above. After selection, cells were grown to confluence in 6 cm dishes. Dishes were transferred on ice, washed once with PBS and lysed in 50 mM Tris, pH 7.4, 150 mM NaCl, 1 mM EDTA, 1% Triton-X, plus protease and phosphatase inhibitors. Samples were clarified by centrifugation (> 10 000 g at 4°C for 10 minutes) and transferred in clean tubes on ice. Protein concentrations were measured using Bradford assay and equalised with lysis buffer. 80 μg of protein samples were set aside as input controls, 600-800 μg of protein were diluted in 200 μL and added to 50 μL of anti-FLAG® M2 affinity gel slurry (Millipore, A2220). Samples were incubated for 1 h rotating at 4°C. Three washes were performed via centrifugation (1000 rpm) using 1 mL of lysis buffer without Triton-X. Elution was performed in 30 μL of wash buffer plus 5 μL of 1 mg/ml 3xFLAG peptide solution (Pierce™ 3x DYKDDDDK, Thermo Scientific, A36805). Samples were incubated on ice for 15 minutes. Beads were pelleted by centrifugation (1000 rpm). 15 μL of eluate were used for western blotting.

### *In vitro* kinase assay

GCN2 KO HeLa cells were transfected with 3xFLAG GCN2 variant constructs as described above. After selection, cells were grown to confluence in 6 cm dishes. Cells were washed once with PBS and incubated with amino acid depleted (lysine and arginine) SILAC medium supplemented with 10% dialysed FBS and 25 mM D-glucose, for 6 h. After immunoprecipitation (as described above) 10 μL of eluate per reaction were added to PCR strip tubes or plates that were previously coated with 100 mg/ml BSA for 3 h, then washed 3X with PBS and dried thoroughly. Kinase assay was performed by adding recombinant eIF2α-NTD, amino acids 2–187 (kindly donated by the Ron lab) to a final concentration on 1 μM and 500 μM ATP in reaction buffer (1X: 50 mM HEPES, pH 7.4, 100 mM potassium acetate, 5 mM magnesium acetate, 250 μg/ml BSA, 10 mM magnesium chloride, 5 mM DTT, 5 mM β-glycerophosphate). Reactions were incubated at 32°C for 10 minutes, then immediately quenched with 5 μL of 6X SDS sample buffer for western blotting, pre-warmed at 95°C.

## Supplementary Material

Figure_S1_4_ddae082

Figure_S2_1_ddae082

Supplementary_legends_SM1_ddae082
