# Supplementary figures and images for: Functional validation of EIF2AK4 (GCN2) missense variants associated with pulmonary arterial hypertension
Source: Hum Mol Genet. 2024 May 22;33(17):1495–505. doi: 10.1093/hmg/ddae082 (PMC11336063; doi:10.1093/hmg/ddae082)

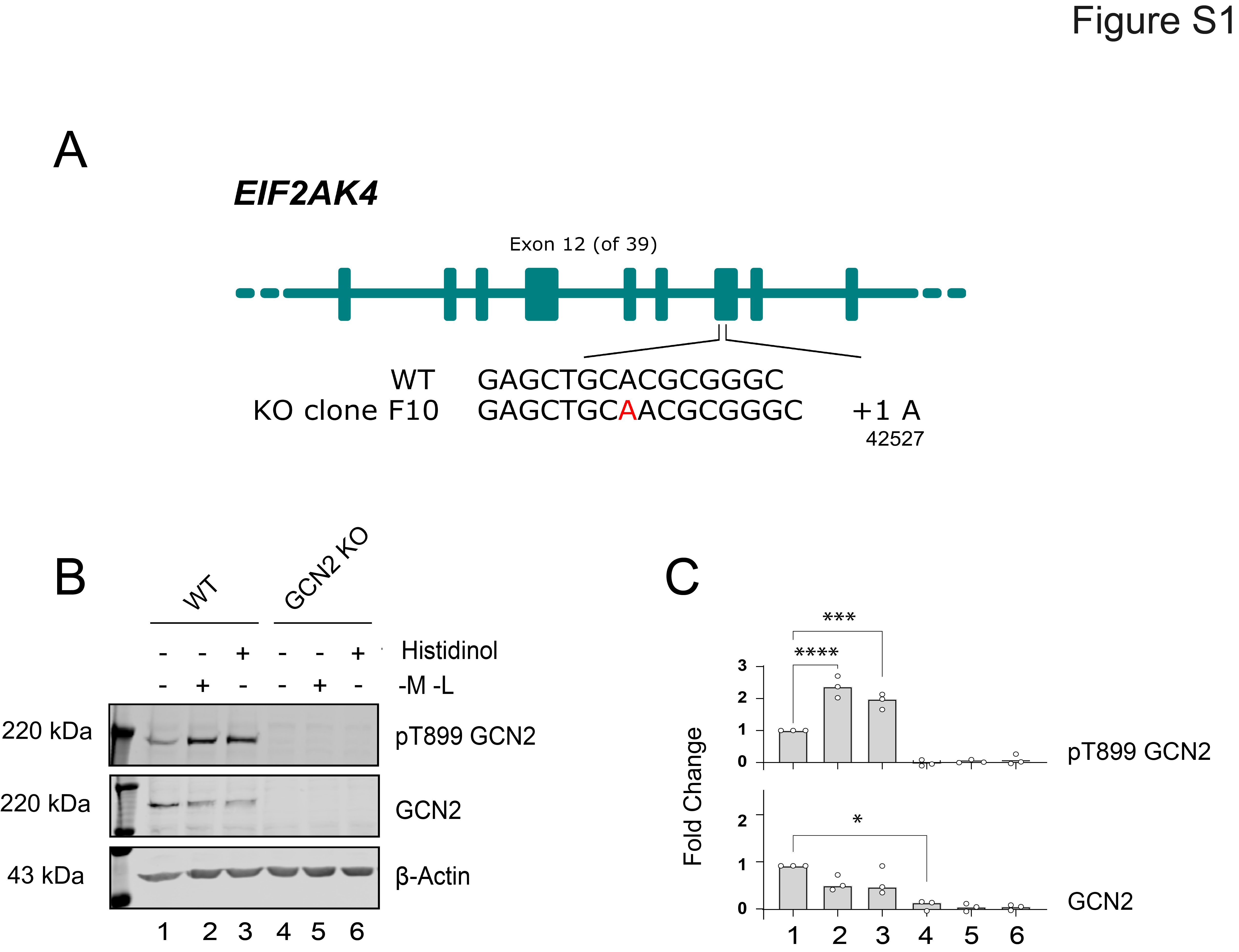

Supplement: Figure_S1_4_ddae082 [file figure_s1_4_ddae082.jpeg]

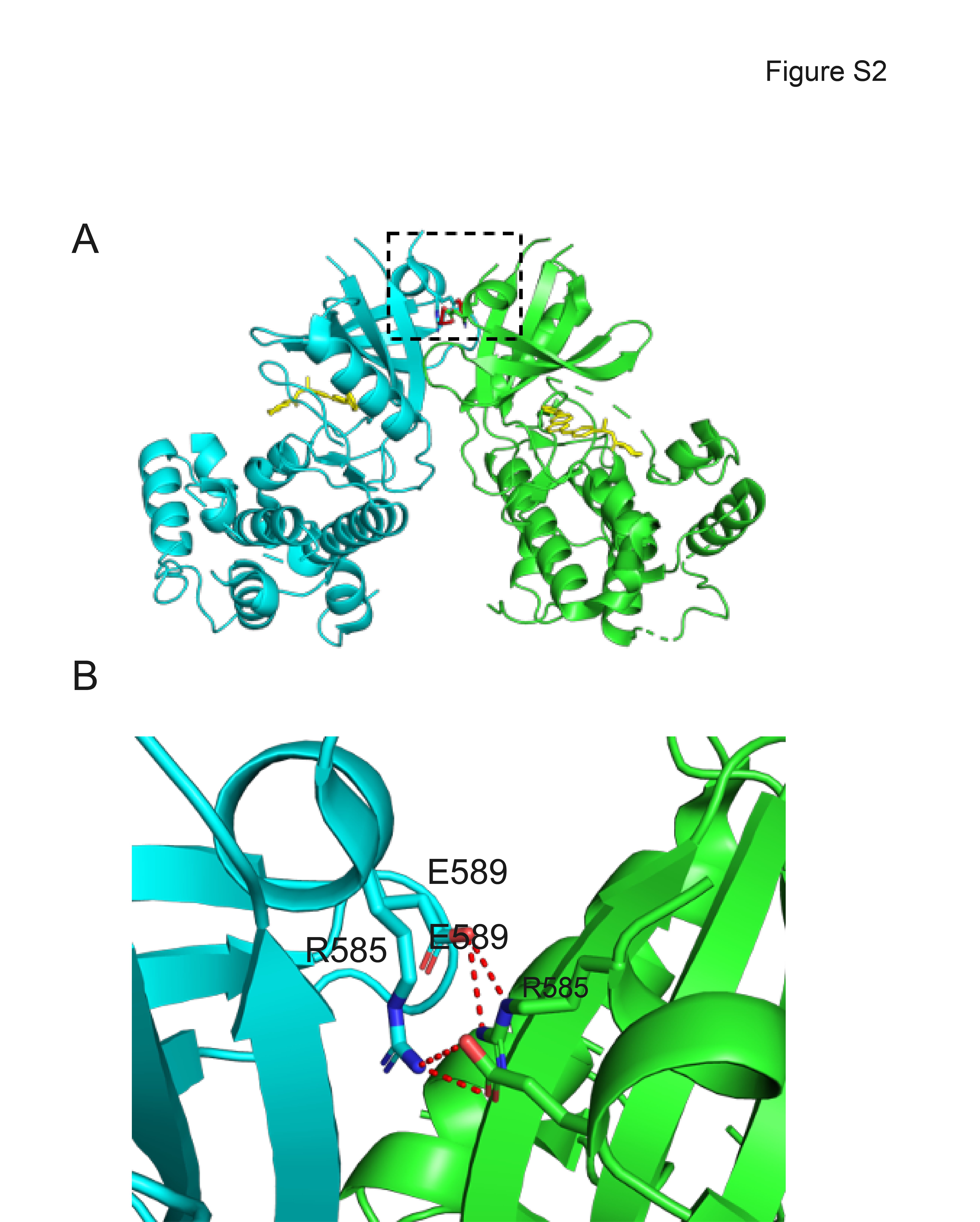

Supplement: Figure_S2_1_ddae082 [file figure_s2_1_ddae082.jpeg]
