# Supplementary material for: Functional validation of EIF2AK4 (GCN2) missense variants associated with pulmonary arterial hypertension
Source: Hum Mol Genet. 2024 May 22;33(17):1495–505. doi: 10.1093/hmg/ddae082 (PMC11336063; doi:10.1093/hmg/ddae082)
Supplement: Supplementary_legends_SM1_ddae082 [file supplementary_legends_sm1_ddae082.docx]

**Supplementary legends**

**Suppl. Figure 1. Knockout of GCN2 in ATF4::nanoLuc reporter HeLa cells**

GCN2 knockout (KO) clonal cell line generated via CrispR/Cas9. (A) Schematic of genomic insertion (homozygous frameshift mutation: +1 alanine insertion at position 42527) in EIF2AK4 generated via CRISPR/Cas9 targeted to exon 12. (B) Representative immunoblot of WT vs GCN2 KO cells treated with 7mM histidinol or starved of methionine and leucine (-M -L) for 7 hours. (C) Quantifications of B; β-actin signal is used as loading control; data in B are normalised to WT untreated (lane 1). n=3 biological replicates. Ordinary 1-way ANOVA with multiple comparisons. * P<0.05, *** P<0.001, **** P<0.0001.

**Suppl. Figure 2. GCN2 kinase domain dimerisation interface**

(A) An overview of the parallel back-to-back GCN2 kinase domain dimer; monomers are in green and cyan (Crystal structure PDB accession: 7QWK)^41^. The box highlights salt-bridges between R585 and E589, reported to stabilise this parallel conformation (active)^38^. (B) Zoomed-in view of the dimer interface, showing the salt bridges between the residues above.
